# (4-Fluoro­phen­yl)[6-(2-fur­yl)-7-nitro-2,3,4,6,7,8-hexa­hydro-1*H*-pyrido[1,2-*a*]pyrimidin-9-yl]methanone

**DOI:** 10.1107/S1600536809026373

**Published:** 2009-07-15

**Authors:** Muhammad Yaqub, Zahid Shafiq, Ashfaq M. Qureshi, Muhammad Najam-ul-Haq

**Affiliations:** a Department of Chemistry, Bahauddin Zakariya University, Multan 60800, Pakistan

## Abstract

In the title compound, C_19_H_18_FN_3_O_4_, the fused pyridine and pyrimidine rings adopt half-chair conformations. The structure displays intra­molecular N—H⋯O and inter­molecular N—H⋯F hydrogen bonding.

## Related literature

For the use of cyclic 1,1-enediamines in the synthesis of a wide variety of fused heterocycles, see: Huang & Wang, (1994 ▶); Yu *et al.* (2006 ▶); Yaqub *et al.* (2008 ▶). For related structures, see: Yu *et al.* (2007 ▶).
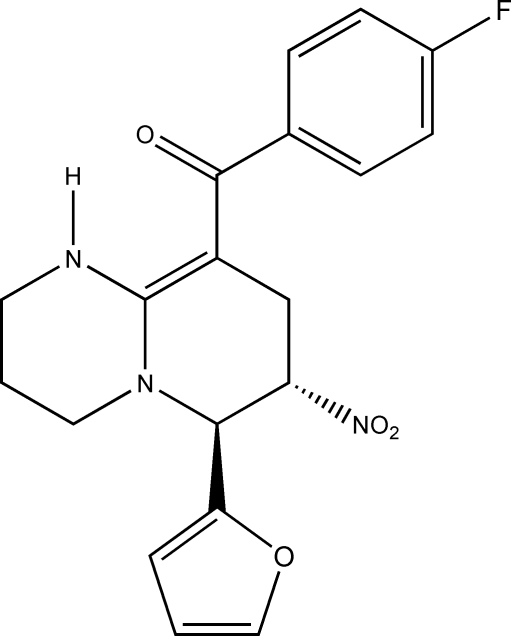


## Experimental

### 

#### Crystal data

C_19_H_18_FN_3_O_4_
*M* *_r_* = 371.36
Orthorhombic, 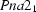

*a* = 15.375 (3) Å
*b* = 7.0706 (14) Å
*c* = 15.255 (3) Å
*V* = 1658.3 (6) Å^3^
*Z* = 4
Mo *K*α radiation
μ = 0.11 mm^−1^
*T* = 173 K
0.38 × 0.25 × 0.19 mm

#### Data collection

Rigaku R-AXIS RAPID IP area-detector diffractometer
Absorption correction: multi-scan (*ABSCOR*; Higashi, 1995 ▶) *T*_min_ = 0.958, *T*_max_ = 0.979
3533 measured reflections
1943 independent reflections
1657 reflections with *I* > 2σ(*I*)
*R*_int_ = 0.020

#### Refinement

*R*[*F* ^2^ > 2σ(*F* ^2^)] = 0.039
*wR*(*F* ^2^) = 0.066
*S* = 1.01
1943 reflections
244 parameters
1 restraint
H-atom parameters constrained
Δρ_max_ = 0.19 e Å^−3^
Δρ_min_ = −0.21 e Å^−3^

### 

Data collection: *RAPID-AUTO* (Rigaku, 2001 ▶); cell refinement: *RAPID-AUTO*; data reduction: *RAPID-AUTO*; program(s) used to solve structure: *SHELXS97* (Sheldrick, 2008 ▶); program(s) used to refine structure: *SHELXL97* (Sheldrick, 2008 ▶); molecular graphics: *SHELXTL* (Sheldrick, 2008 ▶); software used to prepare material for publication: *SHELXL97*.

## Supplementary Material

Crystal structure: contains datablocks I, global. DOI: 10.1107/S1600536809026373/pv2155sup1.cif

Structure factors: contains datablocks I. DOI: 10.1107/S1600536809026373/pv2155Isup2.hkl

Additional supplementary materials:  crystallographic information; 3D view; checkCIF report

## Figures and Tables

**Table 1:** Hydrogen-bond geometry (Å, °)

*D*—H⋯*A*	*D*—H	H⋯*A*	*D*⋯*A*	*D*—H⋯*A*
N1—H1*A*⋯O4	0.88	1.86	2.579 (3)	138
N1—H1*A*⋯F1^i^	0.88	2.60	3.130 (3)	120

Symmetry code: (i) 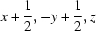.

**Fig. 1.**
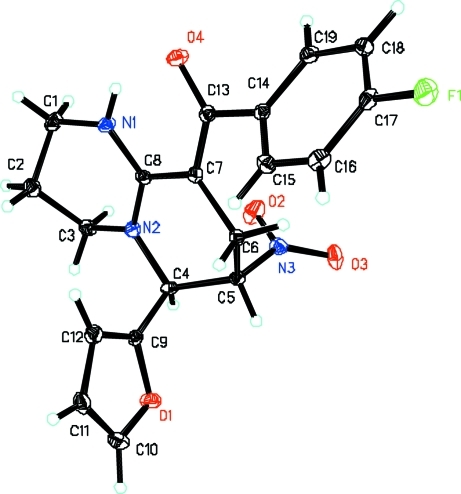
The molecular structure of (I) with displacement ellipsoids drawn at 50% probability level.

**Fig. 2.**
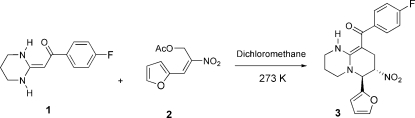
The formation of the title compound.

## supplementary crystallographic information

### Comment

Cyclic 1,1-enediamines known as heterocyclic ketene aminals (HKAs) have been exploited in different synthetic methodologies to build a wide variety of fused heterocycles (Huang & Wang, 1994; Yu, *et al.*, 2006; Yaqub, *et al.*, 2008). The title compound, (I), was prepared by treating nitro derivative of Baylis-Hillman acetates with heterocyclic ketene aminals. The structure of (I) is presented in this article.

The stucture of the title compound, (I), is shown in Fig. 1. The fused pyridyl (N2/C4—C8) and pyrimidyl (N1/N2/C1—C3/C8) rings adopt half-chair conformations, C5 and N2 atoms lie 0.596 (4) and 0.640 (5) Å, respectively, out of the planes formed by the remaining ring atoms. The structure displays an intramolecular (N—H···O) and an intermolecular (N—H···F) hydrogen bonding (details are in Table 1). The molecular dimensions in (I) are in accord with a the corrsponding dimensions reported for a structure very closely related to (I) (Yu, *et al.*, 2007).

### Experimental

(*E*)-2-Nitro-3-(2-furanyl)allyl acetate 2 (0.15 g, 0.71 mmol) and ketene aminal 2 (0.146 g, 0.71 mmol) were stirred in 20 ml of dichloromethane (DCM) at 273 K for one hour. Temperature was allowed to rise up to room temperature and stirring was further continued for 6 hrs. Solvent was evaporated and residue was passed through the column. The elution was carried out by petroleum ether: ethyl acetate (3:1) to get the title compound as a light yellow solid. The single crystals of (I) were grown in dichloromethane - petroleum ether (1:5) system at room temperature by slow evaporation. Yield: 62% (0.16 g), m.p. 417–418 K (lit. m.p. 418–419 K).

### Refinement

An absolute structure could not be established by anomalous dispersion effects because the crystal consists of light atoms only. Therefore, Friedel pairs (1590) were merged. All H atoms were positioned geometrically, with N—H = 0.88 and C—H = 0.95, 0.99 and 1.00 Å, for aromatic, methylene and methine H-atoms, respectively, and constrained to ride on their parent atoms, with *U*_iso_(H) = 1.2*U*_eq_(C, N).

### Crystal data

**Table:**

C_19_H_18_FN_3_O_4_	*F*(000) = 776
*M**_r_* = 371.36	*D*_x_ = 1.487 Mg m^−^^3^
Orthorhombic, *P**n**a*2_1_	Mo *K*α radiation, λ = 0.71073 Å
Hall symbol: P 2c -2n	Cell parameters from 3533 reflections
*a* = 15.375 (3) Å	θ = 2.7–27.4°
*b* = 7.0706 (14) Å	µ = 0.11 mm^−^^1^
*c* = 15.255 (3) Å	*T* = 173 K
*V* = 1658.3 (6) Å^3^	Plate, yellow
*Z* = 4	0.38 × 0.25 × 0.19 mm

### Data collection

**Table:**

Rigaku R-AXIS RAPID IP area-detector diffractometer	1943 independent reflections
Radiation source: rotating anode	1657 reflections with *I* > 2σ(*I*)
graphite	*R*_int_ = 0.020
ω scans at fixed χ = 45°	θ_max_ = 27.4°, θ_min_ = 2.7°
Absorption correction: multi-scan (*ABSCOR*; Higashi, 1995)	*h* = −19→19
*T*_min_ = 0.958, *T*_max_ = 0.979	*k* = −9→9
3533 measured reflections	*l* = −19→19

### Refinement

**Table:**

Refinement on *F*^2^	Primary atom site location: structure-invariant direct methods
Least-squares matrix: full	Secondary atom site location: difference Fourier map
*R*[*F*^2^ > 2σ(*F*^2^)] = 0.039	Hydrogen site location: inferred from neighbouring sites
*wR*(*F*^2^) = 0.066	H-atom parameters constrained
*S* = 1.01	*w* = 1/[σ^2^(*F*_o_^2^) + (0.0225*P*)^2^] where *P* = (*F*_o_^2^ + 2*F*_c_^2^)/3
1943 reflections	(Δ/σ)_max_ < 0.001
244 parameters	Δρ_max_ = 0.19 e Å^−^^3^
1 restraint	Δρ_min_ = −0.21 e Å^−^^3^

### Special details

**Table:**

Geometry. All e.s.d.'s (except the e.s.d. in the dihedral angle between two l.s. planes) are estimated using the full covariance matrix. The cell e.s.d.'s are taken into account individually in the estimation of e.s.d.'s in distances, angles and torsion angles; correlations between e.s.d.'s in cell parameters are only used when they are defined by crystal symmetry. An approximate (isotropic) treatment of cell e.s.d.'s is used for estimating e.s.d.'s involving l.s. planes.
Refinement. Refinement of *F*^2^ against ALL reflections. The weighted *R*-factor *wR* and goodness of fit *S* are based on *F*^2^, conventional *R*-factors *R* are based on *F*, with *F* set to zero for negative *F*^2^. The threshold expression of *F*^2^ > σ(*F*^2^) is used only for calculating *R*-factors(gt) *etc*. and is not relevant to the choice of reflections for refinement. *R*-factors based on *F*^2^ are statistically about twice as large as those based on *F*, and *R*- factors based on ALL data will be even larger.Flack parameter cannot be determined correctly because the crystal consists of light atoms only, and because the radiation is MoKα. In the final stage of structure refinement with SHELXL, MERG 3 card was used, i.e. Friedel pairs (1590) were merged

### Fractional atomic coordinates and isotropic or equivalent isotropic displacement parameters (Å^2^)

**Table:**

	*x*	*y*	*z*	*U*_iso_*/*U*_eq_	
F1	−0.27105 (10)	0.6451 (2)	0.11441 (12)	0.0346 (4)	
O1	−0.16942 (12)	−0.3771 (3)	0.52236 (12)	0.0270 (5)	
O2	0.06546 (13)	0.0877 (3)	0.48554 (15)	0.0412 (6)	
O3	−0.04035 (13)	0.2693 (3)	0.52399 (15)	0.0336 (5)	
O4	0.03402 (12)	0.0794 (3)	0.16274 (12)	0.0249 (4)	
N1	0.08544 (14)	−0.2010 (3)	0.25757 (16)	0.0216 (5)	
H1A	0.0896	−0.1296	0.2105	0.026*	
N2	0.01886 (14)	−0.2546 (3)	0.39083 (16)	0.0188 (5)	
N3	−0.01178 (15)	0.1242 (3)	0.49235 (15)	0.0225 (5)	
C1	0.14602 (18)	−0.3583 (4)	0.2649 (2)	0.0249 (6)	
H1B	0.2037	−0.3107	0.2836	0.030*	
H1C	0.1528	−0.4201	0.2071	0.030*	
C2	0.1128 (2)	−0.5011 (4)	0.3313 (2)	0.0263 (7)	
H2A	0.0632	−0.5723	0.3062	0.032*	
H2B	0.1595	−0.5923	0.3459	0.032*	
C3	0.08430 (18)	−0.3984 (4)	0.41276 (18)	0.0223 (6)	
H3A	0.0594	−0.4898	0.4551	0.027*	
H3B	0.1352	−0.3371	0.4406	0.027*	
C4	−0.04462 (16)	−0.2163 (3)	0.45963 (18)	0.0174 (6)	
H4A	−0.0171	−0.2410	0.5179	0.021*	
C5	−0.07782 (16)	−0.0134 (3)	0.45763 (17)	0.0163 (5)	
H5A	−0.1308	−0.0059	0.4956	0.020*	
C6	−0.10374 (16)	0.0431 (3)	0.36448 (17)	0.0172 (6)	
H6A	−0.1581	−0.0235	0.3484	0.021*	
H6B	−0.1156	0.1806	0.3630	0.021*	
C7	−0.03447 (17)	−0.0028 (4)	0.29753 (17)	0.0177 (5)	
C8	0.02392 (17)	−0.1551 (3)	0.31586 (17)	0.0162 (5)	
C9	−0.12129 (16)	−0.3457 (3)	0.44889 (18)	0.0180 (5)	
C10	−0.24018 (19)	−0.4814 (4)	0.4965 (2)	0.0303 (7)	
H10A	−0.2853	−0.5234	0.5342	0.036*	
C11	−0.23634 (19)	−0.5153 (4)	0.4105 (2)	0.0270 (7)	
H11A	−0.2773	−0.5845	0.3768	0.032*	
C12	−0.15887 (17)	−0.4277 (4)	0.37916 (19)	0.0235 (6)	
H12A	−0.1380	−0.4273	0.3205	0.028*	
C13	−0.02627 (17)	0.0996 (3)	0.21865 (17)	0.0173 (6)	
C14	−0.09337 (16)	0.2471 (3)	0.19533 (18)	0.0170 (6)	
C15	−0.18225 (17)	0.2120 (3)	0.20063 (19)	0.0208 (6)	
H15A	−0.2018	0.0944	0.2234	0.025*	
C16	−0.24299 (18)	0.3450 (4)	0.17349 (19)	0.0240 (6)	
H16A	−0.3036	0.3198	0.1763	0.029*	
C17	−0.21204 (18)	0.5148 (4)	0.14232 (19)	0.0232 (6)	
C18	−0.12529 (18)	0.5575 (4)	0.13609 (18)	0.0236 (6)	
H18A	−0.1065	0.6767	0.1145	0.028*	
C19	−0.06578 (18)	0.4215 (4)	0.16217 (18)	0.0212 (6)	
H19A	−0.0053	0.4472	0.1575	0.025*	

### Atomic displacement parameters (Å^2^)

**Table:**

	*U*^11^	*U*^22^	*U*^33^	*U*^12^	*U*^13^	*U*^23^
F1	0.0346 (10)	0.0353 (9)	0.0338 (10)	0.0157 (8)	−0.0003 (9)	0.0075 (8)
O1	0.0294 (11)	0.0320 (11)	0.0195 (11)	−0.0085 (9)	0.0055 (9)	−0.0003 (9)
O2	0.0187 (11)	0.0488 (14)	0.0560 (16)	−0.0057 (10)	0.0028 (11)	−0.0223 (12)
O3	0.0376 (12)	0.0181 (10)	0.0451 (15)	0.0001 (9)	−0.0059 (11)	−0.0077 (9)
O4	0.0243 (11)	0.0305 (10)	0.0200 (10)	0.0058 (9)	0.0057 (9)	0.0059 (9)
N1	0.0236 (13)	0.0221 (11)	0.0193 (12)	0.0065 (10)	0.0051 (10)	0.0054 (10)
N2	0.0193 (11)	0.0184 (10)	0.0187 (11)	0.0032 (10)	0.0001 (10)	0.0020 (9)
N3	0.0262 (14)	0.0234 (12)	0.0178 (12)	−0.0046 (11)	−0.0004 (10)	0.0000 (10)
C1	0.0230 (14)	0.0254 (14)	0.0263 (15)	0.0098 (13)	0.0049 (13)	−0.0002 (13)
C2	0.0310 (16)	0.0190 (12)	0.0291 (16)	0.0051 (13)	0.0006 (13)	0.0019 (12)
C3	0.0240 (15)	0.0207 (13)	0.0222 (15)	0.0033 (12)	0.0003 (12)	0.0072 (12)
C4	0.0191 (13)	0.0214 (13)	0.0117 (13)	−0.0014 (11)	0.0027 (12)	0.0033 (11)
C5	0.0142 (12)	0.0178 (12)	0.0169 (13)	−0.0022 (11)	0.0006 (11)	−0.0018 (11)
C6	0.0161 (13)	0.0148 (12)	0.0206 (15)	0.0013 (11)	0.0015 (12)	0.0011 (11)
C7	0.0198 (13)	0.0157 (12)	0.0176 (14)	−0.0006 (12)	0.0006 (11)	−0.0014 (10)
C8	0.0154 (13)	0.0165 (12)	0.0167 (13)	−0.0046 (11)	−0.0003 (11)	−0.0011 (11)
C9	0.0216 (13)	0.0151 (12)	0.0173 (13)	0.0035 (11)	0.0029 (12)	0.0015 (11)
C10	0.0283 (17)	0.0289 (15)	0.0338 (19)	−0.0100 (14)	0.0062 (14)	0.0011 (14)
C11	0.0261 (17)	0.0195 (13)	0.0354 (19)	−0.0037 (13)	−0.0020 (14)	−0.0056 (13)
C12	0.0261 (16)	0.0231 (13)	0.0212 (15)	0.0003 (12)	0.0005 (13)	−0.0023 (12)
C13	0.0184 (13)	0.0179 (13)	0.0154 (13)	−0.0012 (11)	−0.0012 (11)	−0.0020 (11)
C14	0.0213 (14)	0.0180 (11)	0.0119 (12)	0.0010 (12)	0.0004 (11)	−0.0020 (10)
C15	0.0242 (14)	0.0189 (13)	0.0194 (14)	−0.0026 (12)	−0.0022 (13)	0.0008 (12)
C16	0.0202 (14)	0.0304 (14)	0.0214 (16)	0.0007 (13)	−0.0016 (12)	−0.0026 (12)
C17	0.0300 (16)	0.0249 (13)	0.0147 (13)	0.0126 (12)	0.0002 (12)	0.0014 (11)
C18	0.0322 (16)	0.0200 (13)	0.0185 (15)	0.0025 (12)	0.0049 (12)	0.0047 (12)
C19	0.0233 (15)	0.0231 (13)	0.0171 (14)	−0.0001 (12)	0.0035 (12)	−0.0002 (12)

### Geometric parameters (Å, °)

**Table:**

F1—C17	1.361 (3)	C5—C6	1.529 (4)
O1—C9	1.361 (3)	C5—H5A	1.0000
O1—C10	1.372 (3)	C6—C7	1.511 (3)
O2—N3	1.220 (3)	C6—H6A	0.9900
O3—N3	1.216 (3)	C6—H6B	0.9900
O4—C13	1.268 (3)	C7—C13	1.410 (4)
N1—C8	1.338 (3)	C7—C8	1.430 (3)
N1—C1	1.455 (3)	C9—C12	1.342 (4)
N1—H1A	0.8800	C10—C11	1.334 (4)
N2—C8	1.345 (3)	C10—H10A	0.9500
N2—C4	1.458 (3)	C11—C12	1.425 (4)
N2—C3	1.469 (3)	C11—H11A	0.9500
N3—C5	1.503 (3)	C12—H12A	0.9500
C1—C2	1.518 (4)	C13—C14	1.509 (4)
C1—H1B	0.9900	C14—C15	1.391 (3)
C1—H1C	0.9900	C14—C19	1.399 (3)
C2—C3	1.505 (4)	C15—C16	1.389 (4)
C2—H2A	0.9900	C15—H15A	0.9500
C2—H2B	0.9900	C16—C17	1.376 (4)
C3—H3A	0.9900	C16—H16A	0.9500
C3—H3B	0.9900	C17—C18	1.371 (4)
C4—C9	1.501 (3)	C18—C19	1.385 (4)
C4—C5	1.523 (3)	C18—H18A	0.9500
C4—H4A	1.0000	C19—H19A	0.9500
			
C9—O1—C10	106.4 (2)	C7—C6—H6B	109.0
C8—N1—C1	125.9 (2)	C5—C6—H6B	109.0
C8—N1—H1A	117.0	H6A—C6—H6B	107.8
C1—N1—H1A	117.0	C13—C7—C8	119.8 (2)
C8—N2—C4	123.6 (2)	C13—C7—C6	122.0 (2)
C8—N2—C3	121.1 (2)	C8—C7—C6	118.2 (2)
C4—N2—C3	115.0 (2)	N1—C8—N2	118.6 (2)
O3—N3—O2	124.4 (2)	N1—C8—C7	119.8 (2)
O3—N3—C5	116.2 (2)	N2—C8—C7	121.6 (2)
O2—N3—C5	119.4 (2)	C12—C9—O1	110.4 (2)
N1—C1—C2	110.2 (2)	C12—C9—C4	133.5 (3)
N1—C1—H1B	109.6	O1—C9—C4	115.9 (2)
C2—C1—H1B	109.6	C11—C10—O1	110.1 (3)
N1—C1—H1C	109.6	C11—C10—H10A	124.9
C2—C1—H1C	109.6	O1—C10—H10A	124.9
H1B—C1—H1C	108.1	C10—C11—C12	106.8 (3)
C3—C2—C1	109.1 (2)	C10—C11—H11A	126.6
C3—C2—H2A	109.9	C12—C11—H11A	126.6
C1—C2—H2A	109.9	C9—C12—C11	106.3 (3)
C3—C2—H2B	109.9	C9—C12—H12A	126.8
C1—C2—H2B	109.9	C11—C12—H12A	126.8
H2A—C2—H2B	108.3	O4—C13—C7	125.6 (2)
N2—C3—C2	110.2 (2)	O4—C13—C14	114.8 (2)
N2—C3—H3A	109.6	C7—C13—C14	119.7 (2)
C2—C3—H3A	109.6	C15—C14—C19	118.4 (2)
N2—C3—H3B	109.6	C15—C14—C13	122.3 (2)
C2—C3—H3B	109.6	C19—C14—C13	119.1 (2)
H3A—C3—H3B	108.1	C16—C15—C14	121.5 (2)
N2—C4—C9	109.5 (2)	C16—C15—H15A	119.2
N2—C4—C5	112.6 (2)	C14—C15—H15A	119.2
C9—C4—C5	108.0 (2)	C17—C16—C15	117.5 (3)
N2—C4—H4A	108.9	C17—C16—H16A	121.3
C9—C4—H4A	108.9	C15—C16—H16A	121.3
C5—C4—H4A	108.9	F1—C17—C18	118.6 (2)
N3—C5—C4	112.1 (2)	F1—C17—C16	117.9 (2)
N3—C5—C6	109.5 (2)	C18—C17—C16	123.5 (3)
C4—C5—C6	110.6 (2)	C17—C18—C19	118.0 (2)
N3—C5—H5A	108.2	C17—C18—H18A	121.0
C4—C5—H5A	108.2	C19—C18—H18A	121.0
C6—C5—H5A	108.2	C18—C19—C14	121.0 (2)
C7—C6—C5	112.9 (2)	C18—C19—H19A	119.5
C7—C6—H6A	109.0	C14—C19—H19A	119.5
C5—C6—H6A	109.0		
			
C8—N1—C1—C2	18.4 (4)	C6—C7—C8—N2	−0.8 (4)
N1—C1—C2—C3	−47.0 (3)	C10—O1—C9—C12	−0.6 (3)
C8—N2—C3—C2	−36.6 (3)	C10—O1—C9—C4	173.9 (2)
C4—N2—C3—C2	149.5 (2)	N2—C4—C9—C12	−30.1 (4)
C1—C2—C3—N2	56.1 (3)	C5—C4—C9—C12	92.8 (3)
C8—N2—C4—C9	96.3 (3)	N2—C4—C9—O1	156.9 (2)
C3—N2—C4—C9	−90.0 (3)	C5—C4—C9—O1	−80.1 (3)
C8—N2—C4—C5	−23.8 (3)	C9—O1—C10—C11	0.5 (3)
C3—N2—C4—C5	149.9 (2)	O1—C10—C11—C12	−0.1 (3)
O3—N3—C5—C4	−153.2 (2)	O1—C9—C12—C11	0.5 (3)
O2—N3—C5—C4	28.6 (3)	C4—C9—C12—C11	−172.7 (3)
O3—N3—C5—C6	83.7 (3)	C10—C11—C12—C9	−0.3 (3)
O2—N3—C5—C6	−94.6 (3)	C8—C7—C13—O4	−5.9 (4)
N2—C4—C5—N3	−74.9 (3)	C6—C7—C13—O4	174.2 (2)
C9—C4—C5—N3	164.1 (2)	C8—C7—C13—C14	173.9 (2)
N2—C4—C5—C6	47.7 (3)	C6—C7—C13—C14	−6.1 (4)
C9—C4—C5—C6	−73.3 (3)	O4—C13—C14—C15	132.0 (3)
N3—C5—C6—C7	74.7 (2)	C7—C13—C14—C15	−47.8 (4)
C4—C5—C6—C7	−49.4 (3)	O4—C13—C14—C19	−44.1 (3)
C5—C6—C7—C13	−153.3 (2)	C7—C13—C14—C19	136.1 (3)
C5—C6—C7—C8	26.7 (3)	C19—C14—C15—C16	0.4 (4)
C1—N1—C8—N2	3.7 (4)	C13—C14—C15—C16	−175.7 (3)
C1—N1—C8—C7	−176.4 (2)	C14—C15—C16—C17	−1.0 (4)
C4—N2—C8—N1	179.2 (2)	C15—C16—C17—F1	179.1 (2)
C3—N2—C8—N1	5.9 (4)	C15—C16—C17—C18	0.7 (4)
C4—N2—C8—C7	−0.7 (4)	F1—C17—C18—C19	−178.0 (2)
C3—N2—C8—C7	−174.0 (2)	C16—C17—C18—C19	0.4 (4)
C13—C7—C8—N1	−0.7 (4)	C17—C18—C19—C14	−1.1 (4)
C6—C7—C8—N1	179.2 (2)	C15—C14—C19—C18	0.7 (4)
C13—C7—C8—N2	179.2 (2)	C13—C14—C19—C18	176.9 (2)

### Hydrogen-bond geometry (Å, °)

**Table:**

*D*—H···*A*	*D*—H	H···*A*	*D*···*A*	*D*—H···*A*
N1—H1A···O4	0.88	1.86	2.579 (3)	138
N1—H1A···F1^i^	0.88	2.60	3.130 (3)	120

Symmetry codes: (i) *x*+1/2, −*y*+1/2, *z*.
